# Downregulation of peroxisome proliferator-activated receptors (PPARs) in nasal polyposis

**DOI:** 10.1186/1465-9921-6-132

**Published:** 2005-11-07

**Authors:** Lars-Olaf Cardell, Magnus Hägge, Rolf Uddman, Mikael Adner

**Affiliations:** 1 Laboratory of Clinical and Experimental Allergy Research, Department of Otorhinolaryngology, Lund University, Malmö University Hospital, Malmö, Sweden

## Abstract

**Background:**

Peroxisome proliferator-activated receptor (PPAR) α, βδ and γ are nuclear receptors activated by fatty acid metabolites. An anti-inflammatory role for these receptors in airway inflammation has been suggested.

**Methods:**

Nasal biopsies were obtained from 10 healthy volunteers and 10 patients with symptomatic allergic rhinitis. Nasal polyps were obtained from 22 patients, before and after 4 weeks of local steroid treatment (fluticasone). Real-time RT-PCR was used for mRNA quantification and immunohistochemistry for protein localization and quantification.

**Results:**

mRNA expression of PPARα, PPARβδ, PPARγ was found in all specimens. No differences in the expression of PPARs were obtained in nasal biopsies from patients with allergic rhinitis and healthy volunteers. Nasal polyps exhibited lower levels of PPARα and PPARγ than normal nasal mucosa and these levels were, for PPARγ, further reduced following steroid treatment. PPARγ immunoreactivity was detected in the epithelium, but also found in smooth muscle of blood vessels, glandular acini and inflammatory cells. Quantitative evaluation of the epithelial immunostaining revealed no differences between nasal biopsies from patients with allergic rhinitis and healthy volunteers. In polyps, the PPARγ immunoreactivity was lower than in nasal mucosa and further decreased after steroid treatment.

**Conclusion:**

The down-regulation of PPARγ, in nasal polyposis but not in turbinates during symptomatic seasonal rhinitis, suggests that PPARγ might be of importance in long standing inflammations.

## Background

Seasonal allergic rhinitis is the result of an immunologically mediated hypersensitivity reaction of the nasal mucosa, initiated by exposure to specific allergens. The reaction is characterized by the infiltration of various inflammatory cells, like eosinophils, neutrophils, basophils, monocytes, and lymphocytes. When the season is over, symptoms and signs of inflammation disappear and the nasal mucosa gradually returns to a situation close to that in healthy non-allergic subjects [1-4]. Nasal polyposis is another inflammatory disorder of the upper airways and is, like allergic rhinitis, related to an infiltration of inflammatory cells [5]. The allergic inflammation is driven by a network of pro-inflammatory cytokines [6] and several of these mediators also appear to be involved in nasal polyposis [7]. In addition, there is an emerging concept that anti-inflammatory pathways affect the outcome of inflammatory diseases, including those in the airways [8]. Peroxisome proliferator-activated receptors (PPARs) have been suggested to play such an anti-inflammatory role [9-11].

Peroxisome proliferator activated receptors (PPARs) are DNA-binding nuclear hormone receptors that are up-regulated in response to high fat diets [12]. PPARs are structurally related to the type II nuclear receptors, including the thyroid hormone receptors and occur intracellularly both in the cytosol and in the nucleus. Although today there are no proven high-affinity pathways for endogenous ligands *in-vivo*, all PPARs are activated by fatty acids. To date, three mammalian PPAR subtypes have been identified, referred to as α, β or δ (here named βδ), and γ, which are encoded by separate genes [13,14]. They share a 60–80% homology in their binding domains and have subtle differences in ligand specificity in that e.g. eocosanoid products of the lipooxygenase pathway such as leukotriene B_4 _and 8-S-hydroxytetraenoic acid (8-S-HETE) activates PPARα [15], prostaglandin (PG) I_2 _activates PPARβδ and, 15-HETE and the PGD_2 _derivative, 15-deoxy-Δ12,14-PGJ_2_, activates PPARγ [16,17]. PPARs are generally expressed at a high level in adipose tissue, and play a prominent role in several physiological processes including the control of lipid and lipoprotein metabolism, and glucose homoeostasis [18]. PPARs might also be involved in cardiovascular disease [19] and cancer [20]. A role for PPARγ in allergic conditions as well as asthma has been suggested, but remains controversial [21]. In murine models of human asthma, it has been demonstrated that local administration of PPARγ agonists decreases serum levels of IgE, and have beneficial effects on airway hyperresponsiveness and lung eosinophilia [22,23]. One study of PPARγ in samples from inflamed human airways has demonstrated that the immunoreactivity for PPARγ is augmented in the bronchial submucosa, the airway epithelium and the smooth muscle cells of asthmatics compared to healthy subjects [24]. The increasing number of reports describing an anti-inflammatory role for PPARs (especially PPARγ) in various disease models [9-11], have prompted us to examine the expression and localization of PPARs in allergic rhinitis and nasal polyposis using nasal polyps as model to investigate the effects of local steroid treatment.

## Methods

### Subjects

The study included 10 patients (five women) with symptomatic birch or grass pollen-induced allergic rhinitis and 10 healthy volunteers (four women), serving as controls. The median age of the patients and controls was 36 (28–50) years and 32 (22–46) years, respectively. In addition, 22 patients with bilateral nasal polyposis (four women) were included before or after treatment with steroids (median age 52 [22–79] years). In 7 of these patients, two sets of polyps were obtained, one before and one after steroid treatment (fluticasone, see below).

The diagnosis of birch and grass pollen induced allergic rhinitis was based on a positive history of intermittent allergic rhinitis and positive skin prick tests to birch and/or timothy. Exclusion criterias included a history of perennial symptoms, upper airway infection during the time of visit, positive skin prick tests to house dust mite (*Dermatophagoides Pteronyssimus *and *D. Farinae*) and molds (*Cladosporium *and *Alternaria*), and treatment with local or systemic corticosteroids during the last 2 months. Occasional use of antihistamines was accepted. The controls were all symptom-free, had no history of allergic rhinitis and had negative skin prick tests to a panel of allergens including birch, timothy, mugwort, house dust mite horse, dog, cat and molds.

Nasal polyposis was identified on the basis of clinical symptoms (nasal obstruction and anosmia) and the visualization of polyps by anterior rhinoscopy. A complete ear, nose-, and throat examination was performed before inclusion. Patients with cystic fibrosis and ciliary dyskinesia were excluded from the study along with subjects with a history of concurrent purulent nasal infection in the six weeks before the study or any kind of nasal surgery during the last year. None of the patients suffered from asthma that required continuous medication.

All patients were recruited through physician referrals and the healthy volunteers were recruited via advertisements in the local press. The study was approved by the Ethics Committee of the Medical Faculty, Lund University.

### Study design for obtaining nasal biopsies

The patients were seen either during the birch (5 patients) or during the grass pollen season (5 patients). They were included when they had experienced substantial symptoms of rhinoconjunctivitis (itchy nose and eyes, sneezing, nasal secretion and nasal blockage) during 3–5 consecutive days. All patients were seen within 5–10 days after the first appearance of symptoms. A local pollen count confirmed the presence of relevant pollen during this period. During the visit patients were asked to evaluate their nasal symptoms, itching/sneezing, secretion and blockage, individually using an arbitrary scale from 0 to 3 (0 = no, 1 = mild, 2 = moderate, 3 = severe symptoms) A total nasal symptom score was then calculated by addition of the three scores. Anterior rhinoscopy was performed and oedema and secretion in each nostril were scored from 0–2 (0 = no, 1 = mild, 2 = severe). Total oedema/secretion scores were then computed by adding the scores for each sign from each nostril. All participants had at least 6 in total symptom score and at least in 5 total oedema/secretion score. The healthy volunteers were seen during the same period.

Biopsies were taken from the inferior turbinate after topical application of local anesthesia containing lidocainhydrochloride-nafazoline for 20 minutes. The specimens for mRNA extraction were immediately placed in RNA-later (QIAGEN) and frozen. For immunohistochemistry, specimens were immersed in an ice-cold fixative solution composed of 2% formaldehyde and 0.2% picric acid, buffered to pH 7.2 with 0.1 M phosphate buffer.

### Study design for obtaining nasal polyps

Patients with nasal polyposis participated in the study during the autumn/winter (outside the pollen season). In patients supplying polyps before treatment all steroids (systemic, inhaled and intranasal) were withheld during a minimum of six weeks before the study (three patients were steroid naïve). All patients supplying polyps after treatment were seen by the surgeon and medication with fluticasone, 200 μg twice a day initiated. After four weeks on this course one set of polyps was removed. Polyps were removed using topical application of local anesthesia containing lidocainhydrochloride-nafazoline for about 20 minutes. In patients providing two sets of polyps a washing out period of two weeks were used after the first set was removed and the Fluticasone medication started.

### RNA extraction and RT-PCR

RNA was extracted from homogenized biopsies using the RNeasy Mini Kit (QIAGEN GmbH), according to the supplier's protocol including an optional DNaseI (Qiagen) treatment. Total RNA quantity and quality were assessed by a spectrophotometer and the wavelength absorption ratio (260/280 nm) was between 1.8 and 2.0 in all preparations. Reverse transcription to cDNA was carried out with Omniscript™ reverse transcriptase kit (QIAGEN GmbH) with oligo-dT primer in a final volume of 20 μl using the Mastercycler personal PCR machine (Eppendorf AG, Germany), at 37°C for 1 h.

### Quantitative real time-PCR

Quantitative real-time PCR assays were performed using the Smartcyckler II detection system (Cephied, USA). Intron over-spanning oligonucleotide primers for detection of PPARα, PPARβδ, PPARγ and β-actin were designed using Primer Express^® ^2.0 software (Applied Biosystem, USA) and synthesized by DNA Technology A/S (Aarhus, Denmark, table 1). PCR was performed using QuantiTect SYBR^® ^Green RT-PCR kit (QIAGEN) in a final volume of 25 μl. Reactions were incubated at 95°C for 15 min, then incubated 46 cycles at 94°C for 30 s followed by 55°C for 60 s (initially 65°C, followed by a 2°C decrease of the first 6 cycles). Standard curves for the PCR reactions were prepared using half ^10^log dilutions of PCR product generated from target cDNA. Specific PCR products were analysed by running melting curve and visualized by agarose electrophoresis.

**Table 1:** Intron over-spanning oligonucleotide primers

**Target**	**Accession nr**	**Primer**	**Sequence (5'-3')**
PPARα	NM005036	forward	ACTCAACAGTTTGTGGCAAGACA
		reverse	GGAAGCACGTCCTCACATGA
PPARβδ	NT007592	forward	GCACATCTACAATGCCTACCTGAA
		reverse	CTCGATGTCGTGGATCACAAA
PPARγ	NM005037	forward	AAGTTCAATGCACTGGAATTAGATGA
		reverse	TGTAGCAGGTTGTCTTGAATGTCTTC
β-actin	NM001101	forward	GCCAACCGCGAGAAGATG
		reverse	ACGGCCAGAGGCGTACAG

Gene expression changes were assessed using the comparative cycle threshold (Ct) method . The relative amounts of mRNA for PPARα, PPARβδ and PPARγ were determined by subtracting Ct values for these genes from the β-actin Ct value (housekeeping gene) and expressed as the amount of mRNA in relation to 100,000 mRNA molecules of β-actin (100,000·2^ΔCt^).

### Immunohistochemistry

The sections from nasal biopsies and nasal polyps were processed for the immunocytochemial demonstration of PPARγ. The PPARγ antibody (Cayman Chemical Company, Ann Arbor, Mi, USA) was raised in rabbit against a peptide corresponding to amino acids 82–101 of human PPARγ1. It cross-reacts with PPARγ2. The antibody was used in dilution 1:800. For the demonstration of the antigen-antibody reaction indirect immunofluorescence was used. Briefly, the cryostat sections were first washed with PBS and then rinsed in PBS for 15 min followed by incubation for 45 min with secondary antibodies raised against rabbit IgG and conjugated to FITC (1:80; swine anti-rabbit FITC, DAKO, Copenhagen, Denmark). Slides were cover-slipped in glycerol/PBS 2:1 (v/v) containing DAP 1 (1 mg/μL) and observed under microscope with chromefluorescence filters.

Negative controls for non-specific binding included normal rabbit serum without primary antibody and secondary antibody alone. Since cross-reactions with other proteins containing amino acid sequences recognized by the antisera could not be excluded, it is appropriate to refer to the immunoreactive material as "PPARγ-like". For brevity, the immunoreactive material is referred to as PPARγ in the text. For quantification, sections were analyzed with Visiopharm Integrator System^® ^v2.1.2 (Visiopharm, Hørsholm, Denmark). The whole batch was immunostained and processed at the same time and the slides were analysed at the same time. Since the background level can differ between the specimens, each slide was individually analysed in that the background level was used as a reference to the induced immunostaining. An intensity reaching a certain threshold was regarded as positive and the area of this staining was measured in relation to the length of the epithelium. The computer program does not analyse the intensity of the staining but only staining that reached a certain level of intensity.

### Statistical analysis

All data sets were analysed by Kolmogorov Smirnov test and since the data for PCR expression predominantly not was Gaussian distributed, Kruskal-Wallis test or Wilcoxon signed rank test was performed and expressed as median value (minimum-maximum), whereas the immunohistochemistry data that were found Gaussian distributed were analyzed by t-test and expressed as mean value ± s.e.m. The null hypothesis was rejected at P < 0.05.

## Results

The standard curves for PPARα, PPARβδ, PPARγ and β-actin had correlation coefficients ranging between 0.93 and 0.99 and generated slope values not significantly different from each other. The efficiency of the PCR reaction was calculated and ranged between 1.96 and 2.01. The RT-PCR analysis of total RNA extracted from nasal biopsies and nasal polyps demonstrated the presence of PPARα, PPARβδ, PPARγ and β-actin in all samples. Melting curve analysis revealed a single peak in each sample and agarose electrophoresis generated expected PCR products with a single band close to the 100 bp marker.

**Figure 1:**
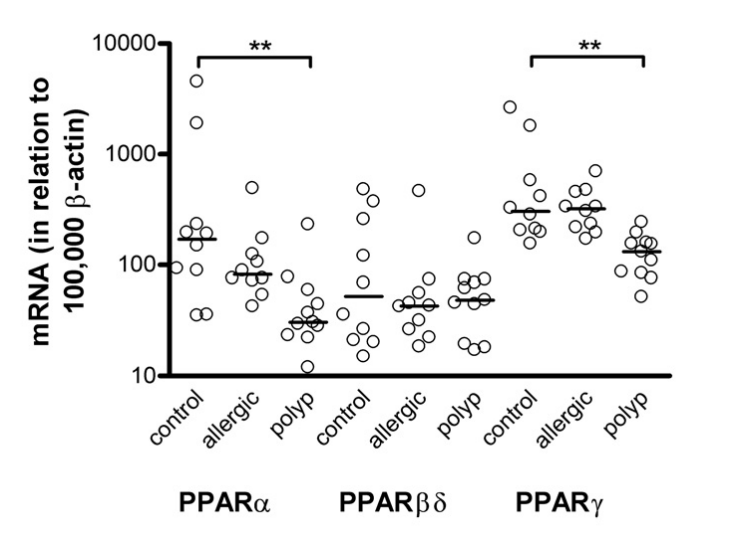
Expression levels of PPARα, PPARβδ and PPARγ in biopsies of the nasal mucosa from 10 healthy volunteers (control) and 10 patients with symptomatic allergic rhinitis (allergic) together with biopsies of nasal polyps from 11 patients with bilateral nasal polyposis (polyp). Levels of PPAR mRNA are calculated in relation to 100,000 mRNA molecules of β-actin. Bold lines represent the median values. The expression of PPARα and PPARγ was lower in polyps than in normal nasal mucosa (**p < 0.01).

Of the three PPARs, the expression of PPARα and γ were generally higher than the levels of PPARβδ. No differences were obtained when expression levels for the different PPARs in nasal biopsies from healthy volunteers were compared with biopsies derived from patients with symptomatic allergic rhinitis (Figure 1): PPARα (mRNA in relation to 100,000 mRNA molecules of β-actin) 170 (53–4512) and 82 (43–491), PPARβδ 52 (14–481) and 43 (18–464) and PPARγ 305 (144–2628) and 321 (171–699) in controls and patients with rhinitis, respectively.

All three PPARs were also detected in nasal polyps obtained from patients not subjected to steroid treatment (Figure 1). The mRNA levels for PPARα and PPARγ were significantly lower in polyps than in normal nasal mucosa (mRNA in relation to 100,000 mRNA molecules of β-actin; 31 (12–232) and 132 (52–243) for PPARα and PPARγ, respectively). No corresponding differences were seen for and PPARβδ

**Figure 2:**
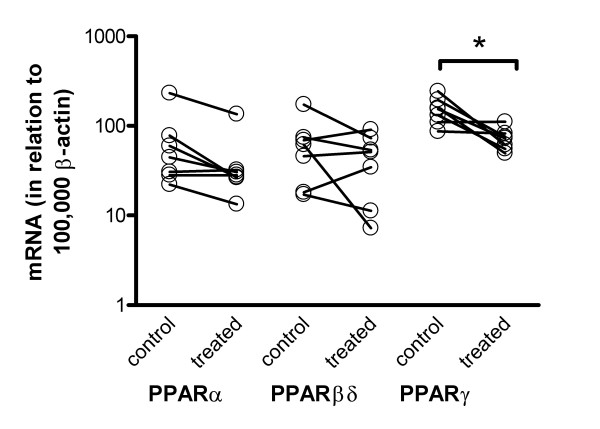
Expression levels of PPARα, PPARβδ and PPARγ in polyps from 7 patients with bilateral nasal polyposis before (control) and after steroid treatment. Levels of PPAR mRNA are calculated in relation to 100,000 mRNA molecules of β-actin. Bold lines represent the median values. The PPARγ levels were reduced following steroid treatment (*p < 0.05).

In order to evaluate if local steroid treatment could affect the expression of the different PPARs we managed to obtain one set before and another set after treatment with steroids from seven of the polyposis patients (Figure 2). Four weeks of treatment resulted in a reduction in the expression of PPARγ (mRNA in relation to 100,000 mRNA molecules of β-actin; 154 (87–244) before and 72 (50–111) after treatment). The expression of PPARα and PPARβδ was not affected by steroid treatment.

**Figure 3:**
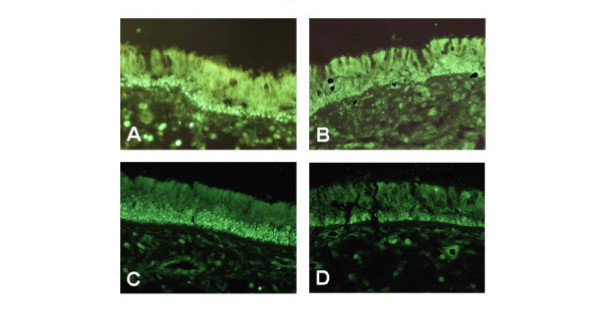
Immunohistochemical localization of PPARγ in biopsies of the nasal mucosa from control subjects (A) and patients with allergic rhinitis (B), and in polyps before (C) and after treatment with steroids (D). Magnification: ×200.

**Figure 4:**
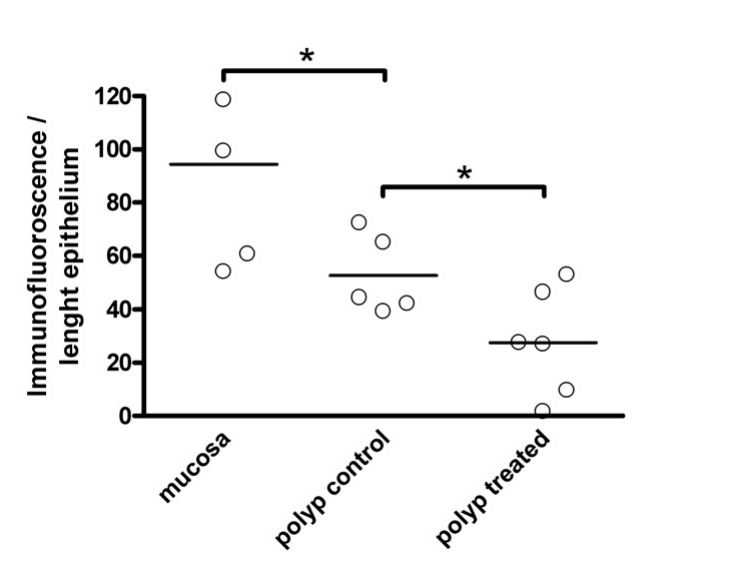
Quantitative computerized analysis of PPARγ immunostained epithelium in sections from biopsies of nasal mucosa and nasal polyps from 5 patients, and 6 patients with fluticasone treatment. The area of those parts of the epithelium that were immunostained by PPARγ antibodies was measured in relation to the length of the epithelium. Bold lines represent the median values. * P < 0.05.

Using immunohistochemistry the protein expression of PPARγ was localized and evaluated (Figure 3A–D). In the nose, PPARγ immunofluorescence was prominent in the surface epithelium, but was also detected in smooth muscle around blood vessels and in acini of small seromucous glands. In addition, PPARγ immunofluorescence was seen in infiltrating inflammatory cells. No differences in PPAR staining could be calculated between nasal biopsies obtained from healthy controls and in biopsies derived from patients with symptomatic allergic rhinitis. In polyps, the PPARγ staining was most prominent in the basal epithelial cells. Quantitative computerized analysis revealed a higher immunoreactivity in the epithelium from biopsies than polyps (area/length units: 94.3 ± 16.4 and 52.6 ± 6.7, respectively; 5 patients from each group; Figure 4). In sections from 5 patients without and 6 patients with steroids, the treatment revealed a reduction after the treatment (area/length units: 52.6 ± 6.7 and 27.5 ± 8.1, respectively).

## Discussion

In the present study mRNA expression of PPARα, PPARβδ, PPARγ was found in all specimens. The expression of PPARs between patients with allergic rhinitis and healthy volunteers was more or less identical. Nasal polyps exhibited lower mRNA expression levels of PPARα and PPARγ than normal nasal mucosa and these levels were, for PPARγ, further reduced following four weeks of treatment with local steroids. PPARγ immunofluorescence was prominent in the epithelium of both normal nasal mucosa and polyps. The epithelial PPARγ immunoreactivity was similar in nasal biopsies from patients with allergic rhinitis and healthy volunteers, but was lower in polyps and further decreased after treatment with fluticasone.

In concordance with the present findings PPARγ has been found to be expressed in cultured human epithelial cells and its activation has been shown to antagonize pro-inflammatory events in this system [25]. In monocytes and macrophages PPARγ-agonists inhibit the expression of proinflammatory cytokines, such as TNF-α, IL-1β, and IL-6 [26,27]. PPARγ is also expressed by eosinophils and agonists inhibit eosinophil chemotaxis and antibody-dependent cellular cytotoxicity reactions *in vitro *[22].

Similar *in vivo *findings have been seen in a murine model of asthma, where treatment with a PPARγ agonist inhibited the development of allergic inflammation, including pulmonary eosinophilia and airway hyperreactivity [22,28]. Furthermore, it was recently demonstrated that cultured human airway smooth muscle cells express PPARα and PPARγ [29], which might relate to the present finding of PPARγ positive cells in conjunction with the vascular smooth muscle in biopsies from both the inferior turbinate and polyps.

Based on animal studies and *in vitro *data, an anti-inflammatory role for PPARγ has been suggested. However, human data to support this idea are still limited. Benayoun and colleagues have shown that PPARγ is augmented in the bronchial submucosa, the airway epithelium, and the smooth muscle of asthmatic patients, as compared with control subjects [24]. The enhanced PPARγ expression is accompanied by increased proliferation and apoptosis of airway epithelial and submucosal cells. In addition, several studies have demonstrated that PPARγ plays an important role in the control of the inflammatory response [21,30], acting on T cells, macrophages, dendritic cells, and mast cells [31-34]. Therefore, an increased expression of PPARγ could have been expected in conjunction with the increased amount of inflammatory cells seen during symptomatic allergic rhinitis. This alteration could not be found in the present study. Since PPARγ appears to be expressed in response to the ongoing inflammation, it might be that it takes more than 3–4 days of pollen exposure to fully activate this putative "defense system".

Nasal polyposis represents a chronic type of inflammation and the lower levels of PPARγ in comparison with normal nasal mucosa might reflect a reduced ability of the diseased mucosa to respond to airway inflammation, thereby facilitating the polyp formation. Steroids (locally administrated, with or without an oral supplement) have, in analogy with our experiments, been reported to down regulate PPARγ expression in bronchial epithelium, mucosa and smooth muscle [24]. Thus, the beneficial effect of glucocorticoid treatment on nasal polyposis may adversely affect the down-regulation of PPARγ. On the other hand, if the inflammatory response is reduced, there will be less need for anti-inflammatory mediators. Notwithstanding whether this is beneficial or not, these studies indicate that PPARγ might be regulated by steroid therapy and that increased knowledge of the physiological effect of PPARγ within the airways might be of importance for our understanding of airway regulation

Neither PPARα nor βδ exhibited any difference in their expression when specimens from healthy volunteers were compared with samples obtained from patients with symptomatic allergic rhinitis. Nor did local steroid treatment affect the expression of these PPARs in nasal polyposis. Inflammation induced by LTB_4_, a PPARα ligand, has been shown to be prolonged in PPARα-deficient mice [15], suggesting an anti-inflammatory role for this receptor. In contrast, in mice injected with lipopolysaccharide (LPS), activation of PPARα induced a significant increase in plasma tumour necrosis factor-α (TNFα) levels [35]. Pro-differentiation and anti-proliferative effects in conjunction with PPARα-stimulation have been demonstrated in various skin models, as well as an ability for this type of stimulation to reduce cutaneus inflammation *in vivo *[36,37]. Albeit the lower expression of PPARα in polyps, the present study did not give any further evidence for a role for PPARα in airway inflammation. For PPARβδ, which is ubiquitously expressed in the human body, the eventual function in inflammation remains uncertain. The relatively low expression level and the unaltered expression seen in the present study add no further information.

The present polyp data could be interpreted as a support for the widespread idea of an anti-inflammatory role for PPARγ within the human airways. However, data contradicting an anti-inflammatory role for PPARγ has been published [38,39]. This discrepancy has been attributed to the use of nonselective ligands [38] or the use of very high concentrations of more selective ligands [39]. In this context, it is essential to recognize that inflammation is normally a self-resolving process with the existence of both positive and negative regulators that ultimately allow complete resolution and homeostasis. In the absence of resolution and clearance or in the event of a dampened healing response, persistent inflammation can arise in the form of tissue damage as associated with chronic disease.

The down-regulation of PPARγ, in nasal polyposis but not in turbinates during symptomatic seasonal rhinitis, suggests that PPARγ might be of importance in long standing inflammations, causing polyps, whereas an eventual role in allergic rhinitis remains to be established. It is tempting to speculate in a therapeutic future for PPARγ activating agonists in the treatment of long standing airway inflammation.

## Competing interests

The author(s) declare that they have no competing interests.

## Authors' contributions

LOC performed the sample preparation and together with MA analyzed the data and drafted the manuscript. RU and MA performed the immunohistochemistry. MH participated in the design of the study and revising the manuscript.
